# Dandy-Walker malformation in methylmalonic acidemia: a rare case report

**DOI:** 10.1186/s12887-021-02874-y

**Published:** 2021-09-13

**Authors:** Jingwei Liu, Zhuohang Liu, Haibo Yan, Yumei Li

**Affiliations:** 1 Department of Pediatric Intensive Care Unit, The First Hospital of Jilin University, Xin Min Street, 130021 Changchun, China; 2 Department of Radiology, The First Hospital of Jilin University, Changchun, China

**Keywords:** Dandy-Walker malformation, methylmalonic acidemia, metabolism disorders

## Abstract

**Background:**

Methylmalonic acidemia is an organic acid metabolism disorder that usually has nonspecific clinical manifestations.

**Case presentation:**

A 3-month-old female infant was admitted to the hospital for developmental retardation. Her prenatal and birth history was unremarkable. After admission, she developed dyspnea and severe anemia and was subsequently transferred to the intensive care unit. Magnetic resonance imaging of her brain showed a Dandy-Walker malformation, and metabolic screening indicated methylmalonic acidemia. Thus, she was diagnosed with methylmalonic acidemia and Dandy-Walker malformation. The patient underwent treatment including acidosis correction, blood transfusion, antibiotics, mechanical ventilation and heat preservation. Unfortunately, her condition progressively worsened and she died of metabolic crisis.

**Conclusions:**

Dandy-Walker malformation may be a clinical manifestation of methylmalonic acidemia. Additionally, the co-existence of methylmalonic acidemia and Dandy-Walker malformation may be an uncharacterized syndrome which needs to be studied further.

## Background

Methylmalonic acidemia is one of the most common organic acid metabolism disorders. Deficiency in the enzyme methylmalonyl-CoA mutase or adenosyl-cobalamin synthesis results in inborn errors of metabolism. The clinical manifestations of patients with methylmalonic acidemia are usually nonspecific. Patients with severe methylmalonic acidemia are prone to lethargy, vomiting, respiratory distress, hypothermia, severe ketoacidosis, and hyperammonemia [1]. This condition can impact many parts of the body, and patients commonly display neurological symptoms. The radiological manifestations of methylmalonic acidemia are also nonspecific [2]. Dandy-Walker malformation is a rare neurodevelopmental anomaly, characterized by cystic dilatation of the fourth ventricle, enlargement of the posterior fossa, hypoplasia of the cerebellar vermis, elevated tentorium cerebelli, and hydrocephalus [3, 4]. Dandy-Walker malformation is a rare imaging manifestation, especially in patients with methylmalonic acidemia.

## Case presentation

**Fig. 1:**
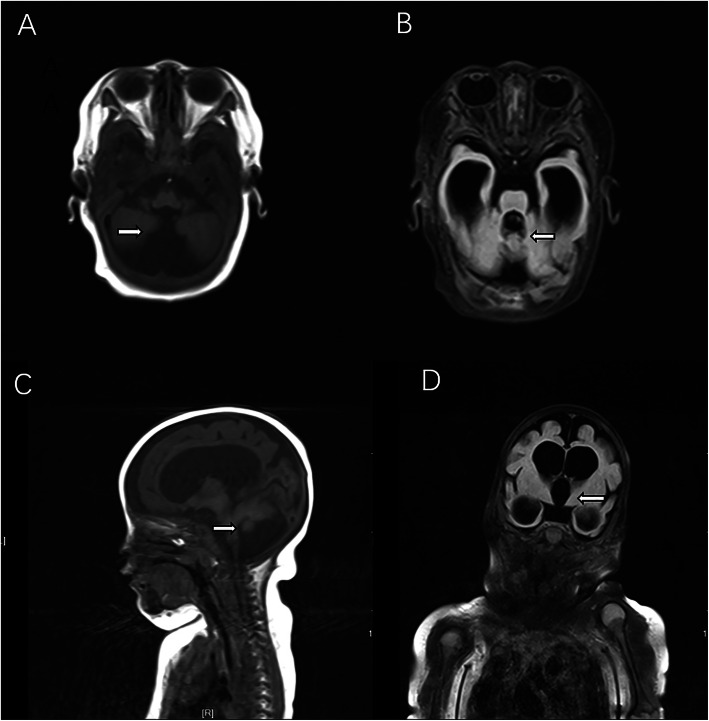
Magnetic resonance imaging of the brain demonstrated cerebellar vermis hypoplasia, enlargement of the fourth ventricle and supratentorial hydrocephalus. Axial T1 weighted imaging (**A**), Axial T2 weighted imaging with fat and fluid suppression (**B**), Sagittal T1-weighted imaging (**C**), Coronal T2 weighted imaging with fat and fluid suppression (**D**)

A 3-month-old female infant was admitted to the hospital for developmental retardation. Her prenatal and birth history was unremarkable. She grew slowly and had a below average height and weight. She could not laugh and raise her head. After admission, she developed dyspnea and severe anemia and was subsequently transferred to the intensive care unit. Auscultation of both lungs indicated some rales. Her lowest body temperature was 34.7℃, and she developed an irregular respiratory rhythm. Since her condition was progressively worsening, she was treated with invasive mechanical ventilation. Routine blood examination showed normal leukocyte and platelet counts, an erythrocyte count of 1.04 × 10^12^/L, and a hemoglobin level of 33 g/L. After a blood transfusion, her hemoglobin gradually increased to a normal level. Blood gas analysis revealed an elevated lactic acid level of 14.62 mmol/L, a decreased pH of 6.84, partial pressure of carbon dioxide of 74.70 mmHg, partial pressure of oxygen of 21.50 mmHg, and potassium level of 6.3 mmol/L. Her C-reactive protein level was 18.40 mg/L. A chest radiograph showed a patchy dense shadow in both lungs. She was also positive for cytomegalovirus immunoglobulin M antibody. Magnetic resonance imaging of her brain showed cerebellar vermis hypoplasia, enlargement of the fourth ventricle, and supratentorial hydrocephalus (Fig. 1). In consultation with the neurosurgery department, it was determined that there was no need for surgical treatment for the time being. The patient’s guardian refused to complete genetic and chromosome analysis. Although the patient received therapy. including acidosis correction with sodium bicarbonate, blood transfusion, antibiotics, mechanical ventilation, and heat preservation, she died 4 days after admission due to metabolic crisis. After her death metabolic screening showed elevated methylmalonic acid and methyl citrate levels.

## Discussion and conclusion

Here, we report a case of methylmalonic acidemia in a patient with a Dandy-Walker malformation. To the best of our knowledge, this brain imaging finding is very rare in patients with methylmalonic acidemia. Most cases of Dandy-Walker malformations are reported in infants below the age of 1 year [5], although sometimes, diagnosis may also be delayed until adulthood [6]. This malformation affects approximately 1/25,000–30,000 newborns [7], and some patients are asymptomatic [6]. Dandy-Walker malformation may develop in isolation or occur as part of a chromosomal abnormality or Mendelian disease [8]. Developmental arrest of the hindbrain before the third month is the most convincing etiological theory for Dandy-Walker malformation. Chromosomal abnormalities and single-gene diseases are the etiologies of Dandy-Walker malformation [9].

Methylmalonic acidemia is a severe and even life-threatening metabolic disease. Methylmalonic acid is produced during the metabolism of some odd-chain fatty acids and amino acids. Methylmalonic acidemia is caused by defects in methylmalonyl-CoA mutase or its coenzyme [2]. The incidence rate of methylmalonic acidemia varies from region to region. One study showed that the incidence of methylmalonic acidemia was 1/51,100 [10]. This disease can involve many parts of the body. Some complications of methylmalonic acidemia include chronic kidney disease [11], diffuse lung disease secondary to methylmalonic acidemia [12], and pulmonary artery hypertension [13, 14]. Pancytopenia and bone marrow hypoplasia may also occur in patients with methylmalonic acidemia during metabolic crises, and this phenomenon correlates with the organic acid concentration [15]. Cerebral and cerebellar atrophy, hypomyelination, reactive gliosis, depletion or hypoplasia of extracerebellar granulosa cells, and multifocal cerebellar hemorrhage are all neuropathologic changes observed in children with methylmalonic acidemia [2]. The main magnetic resonance imaging manifestations of patients with methylmalonic acidemia include cerebral atrophy, white matter abnormalities, ventricular dilatation, and corpus callosal thinning [16]. Our patient developed anemia, metabolic acidosis, respiratory failure, hyperlactatemia, pneumonia, and hypothermia associated with methylmalonic acidemia.

Our patient was positive for cytomegalovirus immunoglobulin M antibody. We are unsure whether the cytomegalovirus infection was congenital due to the lack of cytomegalovirus testing during the neonatal period. The patient had never received anti-cytomegalovirus treatment. Congenital cytomegalovirus infection usually leads to neurologic conditions in children [17]. Prenatal cytomegalovirus infection is a secondary condition of cerebellar hypoplasia [18]. One limitation of this case is that we lack whole exome sequencing and chromosomal “analysis, which may have helped explain the presence of both methylmalonic acidemia and Dandy-Walker malformation in this patient.

Our case highlights a rare association between methylmalonic acidemia and Dandy-Walker malformation. The co-existence of Dandy-Walker malformation and methylmalonic acidemia may be a yet uncharacterized syndrome. It is possible that Dandy-Walker malformation is just a special manifestation of methylmalonic acidemia or alternately was caused by a congenital infection in our patient with methylmalonic acidemia. Regardless, this association merits further study.

## Data Availability

Data sharing is not applicable to this article as no datasets were generated or analyzed during the current study. Data are available from the corresponding author upon request.

## Abbreviations

IgM: Immunoglobulin M
CoA: coenzyme A
